# ROS generation via NOX4 and its utility in the cytological diagnosis of urothelial carcinoma of the urinary bladder

**DOI:** 10.1186/1471-2490-11-22

**Published:** 2011-10-28

**Authors:** Keiji Shimada, Tomomi Fujii, Satoshi Anai, Kiyohide Fujimoto, Noboru Konishi

**Affiliations:** 1 Department of Pathology, Nara Medical University School of Medicine, 840 Shijo-cho, Kashihara city, Nara 634-8521 Japan; 2 Department of Urology, Nara Medical University School of Medicine, 840 Shijo-cho, Kashihara city, Nara 634-8521 Japan

## Abstract

**Background:**

Reactive oxygen species (ROS) production via NADPH oxidase (NOX) contributes to various types of cancer progression. In the present research, we examined the pathobiological role of NADPH oxidase (NOX)4-mediated generation of reactive oxygen species (ROS) in urothelial carcinoma (UC) of the urinary bladder, and demonstrated the utility of ROS labeling in urine cytology.

**Methods:**

NOX4 gene was silenced *in vivo *and *in vitro *by NOX4 siRNA transfection with or without atlocollagen. Cell cycle and measurement of ROS were analyzed by flowcytometry. Orthotopic implantation animal model was used *in vivo *experiment. NOX4 expression in urothelial carcinoma cells was observed by immunohistochemical analysis using surgical specimens of human bladder cancer. Urine cytology was performed after treatment with ROS detection reagents in addition to Papanicolaou staining.

**Results:**

NOX4 was overexpressed in several UC cell lines and the NOX inhibitor, diphenylene iodonium reduced intracellular ROS and induced p16-dependent cell cycle arrest at the G1 phase. Moreover, silencing of NOX4 by siRNA significantly reduced cancer cell growth *in vivo *as assessed in an orthotopic mouse model. Immunohistochemistry demonstrated high expression of NOX4 in low grade/non-invasive and high grade/invasive UC including precancerous lesions such as dysplasia but not in normal urothelium. Then, we assessed the usefulness of cytological analysis of ROS producing cells in urine (ROS-C). Urine samples obtained from UC cases and normal controls were treated with fluorescent reagents labeling the hydrogen peroxide/superoxide anion and cytological atypia of ROS positive cells were analyzed. As a result, the sensitivity for detection of low grade, non-invasive UC was greatly increased (35% in conventional cytology (C-C) vs. 75% in ROS-C), and the specificity was 95%. Through ROS-C, we observed robust improvement in the accuracy of follow-up urine cytology for cases with previously diagnosed UC, especially in those with low grade/non-invasive cancer recurrence (0% in C-C vs. 64% in ROS-C).

**Conclusions:**

This is the first report demonstrating that ROS generation through NOX4 contributes to an early step of urothelial carcinogenesis and cancer cell survival. In addition, cytology using ROS labeling could be a useful diagnostic tool in human bladder cancer.

## Background

Urinary bladder cancer is the seventh most common cancer in the world, with over 10,000 new cases diagnosed annually in Japan. At initial diagnosis, 75% of cases present as superficial (pTa and pT1) tumors and have a good clinical outcome (5-year survival rates ranging from 88% to 98%). However, approximately 50% of patients with non-muscle invasive tumors will experience disease recurrence within 4 years of their initial diagnosis, and 11% will progress to the invasive phenotype [1]. The remaining 25% of cases present with muscle invasive bladder cancer and have a poor prognosis (5-year survival rate ranging from 46% to 63%) [2,3].

Other factors that affect the survival of bladder cancer patients include tumor grade, morphological features, and the presence of lymphovascular invasion or node metastasis. Over the years, several variants of bladder cancer have been recognized; for example, some reports described an adverse outcome for urothelial carcinomas with squamous, glandular, or trophoblastic differentiation, or nested variants, or invasive micropapillary variants and so on [4]. In 2004, the World Health Organization published a classification system for tumors of the urinary system with criteria for patient risk stratification. Specific variants have a distinct therapeutic strategy in addition to the conventional clinical management of urothelial carcinoma [5,6].

Overall, not only is early and accurate histological diagnosis and staging important but long-term surveillance is also required for the successful management of human bladder cancer. The current gold standards for diagnosis and surveillance are conventional white light cystoscopy and urine-based cytology. The former enables direct visualization of tumors and histopathological analysis from biopsy samples. However, cystoscopy is a painful procedure that is associated with iatrogenic risk; moreover, small areas of flat tumors as well as carcinoma in situ can easily be missed [7]. In contrast, urine-based cytology is a simple and cost-effective procedure that can detect tumors with high specificity (78-100%) whether they are visualized or not. The sensitivity is low (12.2-84.6%), however, especially for low-grade tumors, which are the most prevalent and subject to considerable interobserver variability [8]. If we can, therefore, identify a solution that compensates for these disadvantages, cytology will be one of the simplest and most reliable diagnostic and screening tools available with high reproducibility.

Reactive oxygen species (ROS) are implicated in both stimulation and inhibition of cell proliferation, apoptosis, and cell senescence [9,10]. ROS trigger genetic programs associated with transformation, resulting in the alteration of genes by manipulating the cell cycle and signal transduction pathways [11]. Similarly, generation of oxygen radicals is increased in Ras-transformed fibroblasts [12] and antioxidants can block DNA synthesis, suggesting involvement of ROS in mitogenic signaling in the course of neoplastic transformation. NADPH oxidase (NOX) is one of the major sources of cellular ROS [13]. NOX enzymes are the structural homologues of phagocytic NOX (gp91phox/NOX2) and consist of both single (NOX1-NOX5) and dual oxidases (DuOX1 and DuOX2). Emerging evidence suggests that low levels of ROS generated by NOX enzymes act as mediators in inflammation, apoptosis, cell growth, and angiogenesis in various human cancers. Recently, we identified generation of intracellular hydrogen peroxide by NOX1 as a major downstream signal for a novel human AlkB homologue, ALKBH-8, that contributes to cell survival by enhancing resistance to apoptosis in human urothelial carcinoma cells, both in vitro and in vivo [14]. Since hALKBH8 and NOX1 are immunohistochemically overexpressed in high-grade invasive urothelial carcinomas but not in low-grade non-invasive phenotypes, ROS generation through NOX1 plays an important role in the progression of bladder cancer, but not in carcinogenesis or the early stages of cancer development.

In the present study, we show that NOX4 maintained intracellular generation of ROS in both high and low grade and superficial and invasive urothelial carcinoma cells and contributes to cancer cell survival, using in vitro and in vivo experiments and clinicopathological analyses. Moreover, fluorescent labeling of ROS-positive cells in voided urine samples made easy detection of exfoliated cancer cells possible, resulting in improved sensitivity of urine cytology in bladder cancers.

## Methods

### Cell culture, plasmids, and chemicals

The human urothelial carcinoma cell lines, T24, UMUC6, and KK47, were purchased from American Type Culture Collection (Manassas, VA) and cultured in RPMI supplemented with 10% fetal bovine serum. We purchased the anti-p16 antibody from Cell Signaling (MA); anti-actin antibody from Santa Cruz Biotech. Inc. (CA); anti-NOX4 antibody from Abcam (UK) and diphenyleneiodonium (DPI) from Wako (Osaka, Japan).

### Preparation of cell lysates and western blotting analysis

We resolved the cell lysates in sodium dodecyl sulfate (SDS)-polyacrylamide gels and transferred them to polyvinylidene difluoride membranes (Millipore, Ltd.), which were then blocked in 5% skim milk at room temperature for 1 h. We incubated the membranes with the indicated primary antibody for 1 h and then incubated with horseradish peroxidase-conjugated anti-mouse or anti-rabbit IgG (Amersham Pharmacia Biotech). We detected peroxidase activity on X-ray films by using an enhanced chemiluminescence detection system.

### Reverse transcription PCR

Using the OneStep reverse transcription polymerase chain reaction (RT-PCR) kit (QIAGEN), we extracted total RNA by using the Trizol reagent and subjected it to RT-PCR. The PCR conditions were 95°C for 30 s, 55-60°C for 30 s, and 72°C for 1 min through a total of 30 cycles. The PCR primer sequences for NOX2 were 5'-GGGCTGTTCAATGCTTGTGGCT-3' (sense) and 5'-ACATCTTTCTCCTCATCATGGTGC-3'(antisense). The primers for NOX3 were 5'-ATGAACACCTCTGGGGTCAGCTGA-3' (sense) and 5'-GGATCGGAGTCACTCCCTTCGCTG-3' (antisense). The primers for NOX4 were 5'5'-CTCAGCGGAATCAATCAGCTGTG-3' (sense) and 5'- AGAGGAACACGACAATCAGCCTTAG-3' (antisense). The primers for NOX5 were 5'-ATCAAGCGGCCCCCTTTTTTTCAC-3'(sense) and 5'- CTCATTGTCACACTCCTCGACAGC-3' (antisense). The primers used for actin were 5'-ATGGGTCAGAAGGATTCCTATGT-3' (sense) and 5'-GAAGGTCTCAAACATGATCTGGG-3' (antisense).

### Identification and measurement of ROS

We assessed the production of intracellular superoxide anion (O_2_^-^) and H_2_O_2 _with dihydroethidium (DHE, Wako, Osaka, Japan) and 5,6-chloromethyl-2',7'-dichlorodihydrofluorescein diacetate (CM-DCF-DA, Wako), respectively. Cells were incubated with 5 μM DHE or DCF-DA solution for 30 min at 37°C. Excess DHE or DCF-DA was washed, and then superoxide anion (O_2_-) or hydrogen peroxide (H_2_O_2_) formation in the cells was visualized using a Leica CTR6000 photomicroscope and measured by flow cytometry.

### Transfection of NOX4 siRNA

For our transfection analyses, 10^6 ^cells from each urothelial carcinoma cell line were seeded in 6-cm dish plates and transfected with either 100 nM of control RNA (Santa Cruz) or with the small interfering RNA (siRNA) of NOX4. Transfections were carried out using the Lipofectamine system (Invitrogen) in accordance with the manufacturer's protocol. The human NOX4 siRNA duplexes, generated with 3'-dTdT overhangs and prepared by QIAGEN (Hs-NOX4-1-HP siRNA), were chosen against the DNA target sequences as follows: 5'-TCCATTTGCATCAATACTCAA-3'.

### Cell proliferation assay

Cells were stimulated with various reagents for a given period, after which methanethiosulfonate (MTS) reagent (3-(4,5-dimethylthiazol-2-yl)-5-(3-carboxymethoxyphenyl)-2-(4-sulphonyl)-2H-tetrazolium, inner salt; Promega, Tokyo, Japan) was added. After a 3-h incubation period, optical absorbance at 490 nm was measured using a microplate reader. Cell viability was expressed as a mean percentage of the standard deviations of absorbance before and after treatment with various reagents. All experiments were performed in triplicate.

### Tissue samples and immunohistochemistry

We obtained specimens of human urinary bladder cancers diagnosed as urothelial carcinomas (n = 82) from patients undergoing transurethral resection or radical cystectomy without previous history of radiation or chemotherapy at the Nara Medical University Hospital. Clinicopathologic data of the cases were reviewed by 2 urological pathologists (K.S. and N.K., Department of Pathology, Nara Medical University Hospital) and summarized in Table 1. Normal or atypical urothelial tissue samples found in patients with or without severe cystitis (induced by intravesical stones, long-lasting catheterization, etc.) were obtained from autopsy cases (n = 17 for each). The study was approved by the institutional research board of Nara Medical University, and informed consent was obtained from all patients. Tumor stage and grade were noted at the time of diagnosis before the collection of specimens. We followed the same tissue fixation and processing procedure as described in a previous report [15-17]. After deparaffinization, the sections were heated for 5 min in 10 mM of sodium citrate buffer (pH 6.0) in a pressure cooker. The sections were then incubated overnight at 4°C with the indicated antibodies. The reactions were visualized using the Histofine streptavidin-biotin-peroxidase complex (SAB-PO) kit with diaminobenzidine as the chromogen (Nichirei, Tokyo, Japan) and hematoxylin counterstaining. The percentage of cells positive for NOX4 were expressed per 1,000 cells examined.

**Table 1:** Characteristics of urothelial carcinomas for immunohistochemistry

Age (years)	65 (54-83)
Gender (M:F)	61:21
Stage	
pTa	19
pT1	22
≧pT2	14
pTis	27
Grade	
Low grade	25
High grade	57
Total	82

### Conventional and ROS-labeled cytological analyses

We obtained 50 voided urine samples and histological specimens from bladder cancer patients without previous bladder cancer (samples were submitted at the initial diagnosis) and 80 cases without neoplasia (cystitis, bladder stone, chronic kidney disease etc) (Table 2). In addition, 62 follow-up urine samples from patients with previous bladder cancer that were negative (non-recurrence; n = 34) or positive (first to third recurrence; n = 28) were available, in positive cases, and histological specimens were submitted as well (Table 3). In all follow-up cases, the diagnosis was made using histology if a tumor or a questionable lesion (such as redness, erosion, or papillary growing lesions) was found. Voided urine specimens were collected and incubated with 10 μM DHE and CM-DCFDA for detection of superoxide anion (O_2_-) and hydrogen peroxide in serum-free RPMI medium for 30 min at 37°C in the dark. After reaction, the cells were washed with phosphate-buffered saline (PBS) 3 times and immediately fixed in Cytolit, and then Papanicolaou-stained ThinPrep or Cytospin slides were prepared. The diagnosis using Papanicolaou stain samples was made on the basis of 3 categories (negative, suspicious, and positive) by a team of 2 cytopathologists (K.S. and N.K.). Carcinoma was highly suspected, but the number of cytomorphologic changes of the atypical cells was not enough to make a definitive positive diagnosis, for example, due to therapy-mediated cellular degeneration. At the same time, urine samples were examined through a fluorescein isothiocyanate (FITC)-Texas Red (Tx2) filter by fluorescence microscopy. Results were expressed as positive if more than 20 double-positive cells with features of cellular atypia were found in each sample. Once a positive diagnosis was made, the remaining specimen was incubated with the ROS scavenger, *N*-acetyl-L-cysteine, and we checked whether fluorescent reactivity was lost. If fluorescence was not significantly reduced or lost, results were expressed as invalid to preclude autofluorescence. Inflammatory cells also produced ROS, but the level was much lower than that for urothelial carcinoma cells, and these cells were morphologically excluded with Papanicolaou staining. The size of tumor cells was slightly decreased after incubation with ROS detecting reagents in the cell culture medium for 30 min at 37°C. However, cellular atypia, including hyperchromasia, enlarged nucleoli, irregular shape and so on, was not significantly modified; therefore, there was no problem with diagnosis by conventional cytology.

**Table 2:** Characteristics of patients with initial diagnosis of urothelial carcinoma

Age (years)	70 (44-96)
Gender (M:F)	40:10
Stage	
pTa	10
pT1	6
≧pT2	6
pTis	8
Grade	
Low grade	24
High grade	26
Total	50

**Table 3:** Comparison of conventional cytology with ROS cytology (initially diagnosed urothelial carcinomas)

	Conventional cytology				ROS cytology		
Urothelial carcinoma	Positive	Suspicious	Negative	Sensitivity	Positive	Negative	Sensitivity
Low grade(n = 24)	9	7	8	35%	18	6	75%
High grade(n = 26)	21	1	2	88%	22	2	92%
Non-invasive papillary	10	8	8	39%	20	6	77%
(n = 26)							
Superficially invasive	6	1	0	86%	6	1	86%
(n = 7)							
Deep invasive	6	0	0	100%	6	0	100%
(n = 6)							
Carcinoma in situ	8	0	1	89%	8	1	89%
(n = 10)							
				Specificity			Specificity
Negative(n = 80)	6	13	72	90%	4	76	95%

### Orthotropic implantation animal model

Animal experiments were approved by the institutional animal care and use committee at Nara Medical University. Eight week-old female nude mice were maintained on a daily 12-h cycle of light and dark and were fed standard diet and water ad libidum [14,18]. The mice were purchased from CLEA (Tokyo, Japan) and maintained with the CLEA Rodent diet http://www.clea-japan.com/Feed/cl2.html. Fourteen days after transcatheter inoculation of 5 × 10^7 ^KU-7 cells with or without green fluorescent protein (GFP) expression in the urinary bladder, the mice were randomized into either control (control RNA + atelocollagen, n = 10) or NOX4 knockdown (NOX4 siRNA + atelocollagen, n = 8) treatment groups and received a single intravesical treatment instillation that was retained for 1 h by purse-string suture. The mice were sacrificed 14 days after treatment, and the bladders were removed, splayed open on filter paper, and fixed in 10% neutral buffered formalin, or tumor cells were homogenized and treated with CM-DCFDA or DHE, followed by analysis of ROS generation by flow cytometry.

### Image analysis

The tumor burden was determined by analyzing images of GFP fluorescence from tumor cells on flat formalin-fixed bladders captured by a fluorescent stereomicroscope. Image analysis was done with Image J public domain software available through the National Institute of Health. All images were spatially calibrated for area measurements. The mice were then sacrificed, and the bladders were excised and fixed for histologic examination by hematoxylin and eosin stain and immunohistochemistry.

### Statistical analysis

Data were statistically analyzed using the student's *t*-test or non-parametric analysis Kruskal-Wallis test [14,18]. The results were considered significant if P value was less than 0.05.

## Results

### NOX4-mediated generation of ROS affects urothelial carcinoma cell survival in vitro

**Figure 1:**
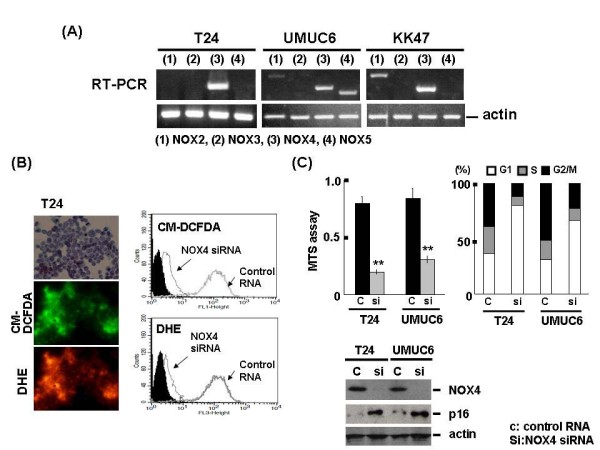
**The role of NOX4-mediated ROS generation in human bladder cancer cells**. (A) RNA was extracted from human urothelial carcinoma cell lines, T24, UMUC6 and KK47, then mRNA levels of NOX 2, 3, 4 and 5 were analyzed by RT-PCR. mRNA expression of actin was used for (B) T24 cells were treated by ROS detecting fluorescence reagents, CM-DCFDA and DHE, and ROS generation was assessed by fluorescence microscopy (left panel). T24 cells were tarnsfected by NOX4 siRNA at 100 nM and control RNA. After 72 h inoculation, cells were treated with CM-DCFDA and DHE, then intracellular ROS levels were analyzed by flowcytometer as described. Solid black indicates negative control (treated with DMSO). (C) T24 and UMUC6 cells were tarnsfected by NOX4 siRNA at 100 nM and control RNA. After 72 h inoculation, MTS assay, flowcytometric analysis using propidium iodide or western blotting using anti-NOX4, p16 antibody was performed. Actin was used for protein or mRNA expression of housekeeping gene throughout the experiments. Each value is the mean ± SE.

**Figure 2:**
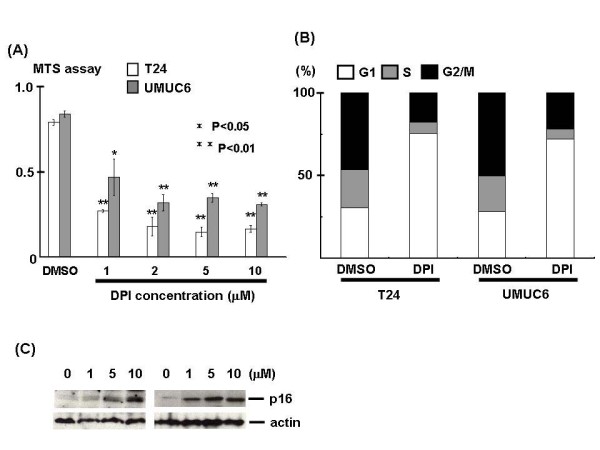
**NOX inhibition induced cell cycle arrest, resulting in suppression of cancer cell growth**. T24 and UMUC6 cells were treated with DPI at the indicated concentrations for 48 h, then MTS assay(A), flowcytometric cell cycle analysis using propidium iodide (B) or western blotting using anti-p16 antibody (C) was performed. Each value is the mean ± SE.

At the beginning of the experiment, the expression of NOX family members, apart from NOX1, was examined in the human urothelial carcinoma cell lines T24, UMUC6, and KK47. RT-PCR analysis showed that NOX4 was highly expressed in all 3 cell lines. ROS, including the radical superoxide anion and non-radical hydrogen peroxide, were constitutively generated as examined by fluorescent microscopy or flow cytometry using DHE and CM-DCF-DA, but ROS levels significantly declined following NOX4 siRNA transfection (Figures 1A and 1B). The same finding was observed in the UMUC6 and KK47 cell lines (data not shown). Moreover, NOX4 knockdown by siRNA transfection suppressed cell growth via p16 induction and cell cycle arrest at the G1 phase in the T24 and UMUC6 cell lines (Figure 1C)). Similarly, treatment with the NOX inhibitor, diphenylene iodonium (DPI), induced p16 and cell cycle arrest at the G1 phase, resulting in inhibition of cancer cell growth in T24 and UMUC6 cells (Figure 2). Treatment with ROS scavengers, such as *N*-acetyl-L-cysteine, induced cell cycle arrest as expected and inhibited the rate of cell growth (data not shown).

### NOX4 gene silencing reduced tumor volume in orthotopic implantation model

**Figure 3:**
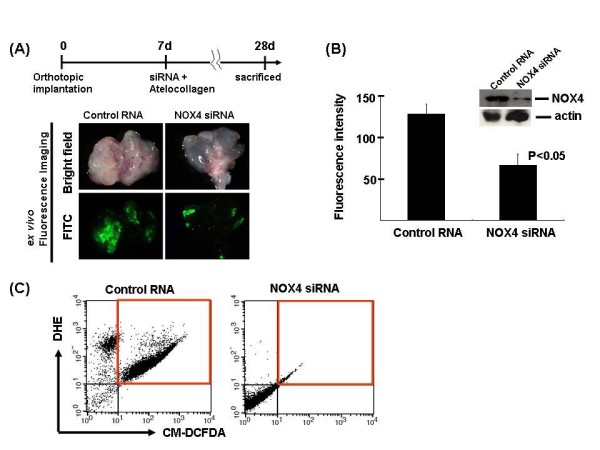
**Intravesical injection of NOX4 siRNA inhibits tumor growth in vivo in the mouse orthotopic bladder cancer implant model**. (A) Upper panel indicates brief experimental protocol. 14 days after KU7/GFP cells were transplanted into the mouse bladder, atelocollagen with either NOX4 siRNA or control RNA was transurethrally instilled into the bladder lumen. Urinary bladders were resected 14 d post-instillation, and in situ images captured under fluorescent light (lower panel). (B) indicates growth suppression in lesions treated with NOX4 siRNA/atelocollagen and control RNA/atelocollagen was compared in terms of tumor area measured by GFP expression and histologic analysis. Columns, mean; bars, SE. Bladder cancer samples were resected, lysed, and expression of NOX4 was determined by Western blot analysis. (C) Bladder cancer samples derived from control and siRNA of NOX4 treatments animals were homogenized and cells were stained by CM-DCFDA and DHE. Then, flowcytometric analysis was performed to determine the level of intracellular hydrogen peroxide/superoxide anion.

We examined the role of NOX4 in bladder cancer development in vivo. Using a KU7 cell line stably overexpressing GFP, we previously established GFP image analysis in this system, so that tumor growth and treatment response can be quickly measured [14]. As shown in Figures 3A and 3B, NOX4 down-regulation (knockdown efficacy was 60-70% as assessed by western blotting) by instillation of siRNA combined with atelocollagen produced a significant decrease in the tumor area in the orthotropic implantation mouse experiment. Flow cytometric analysis indicated that CM-DCFDA and DHE double-positive populations in implanted tumor cells were strongly reduced by NOX4 gene silencing, which was consistent with in vitro experimental data (Figure 3C). These results clearly show that NOX4-dependent ROS generation plays an important role in the growth of urothelial carcinoma cells both in vitro and in vivo.

### NOX-4 is expressed in urothelial carcinoma but not in normal urothelium of human urinary bladder

**Figure 4:**
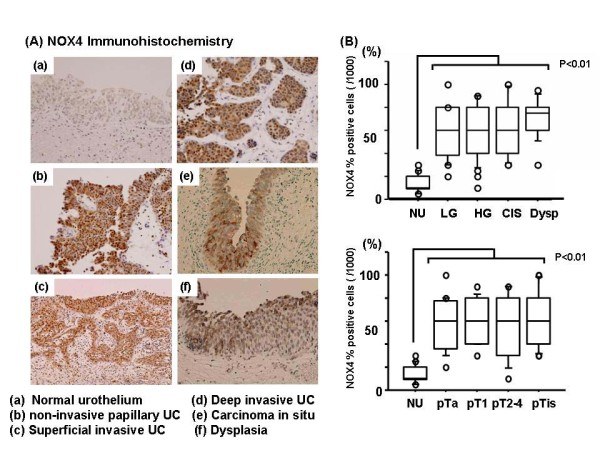
**Immunohistochemical analysis of NOX4**. Immunohistochemistry for NOX4 expression (A). The percentage of immunopositive cells was calculated per 1,000 cells/high-power field (B). Each value is the mean ± SE.

We performed immunohistochemistry for NOX4 in surgical specimens of human bladder cancer and normal tissue derived from autopsy samples (Table 1). Figures 4A-a, b, c, d, e and 4f clearly demonstrate that NOX4 is seldom expressed in normal urothelium, nor is it expressed in reactive atypia (mostly due to chronic inflammation/denudation), but was overexpressed in low or high-grade and non-invasive or invasive urothelial carcinoma cells, including carcinoma in situ (CIS). The NOX4 expression level was not correlated to pathological parameters such as grade, stage, and tumor size (Figure 4B). Interestingly, the immunopositive findings for NOX4 for the precancerous lesion dysplasia were statistically higher than those for normal urothelium.

### The use of ROS labeling in urine cytology for bladder cancer (initially diagnosed cases)

**Figure 5:**
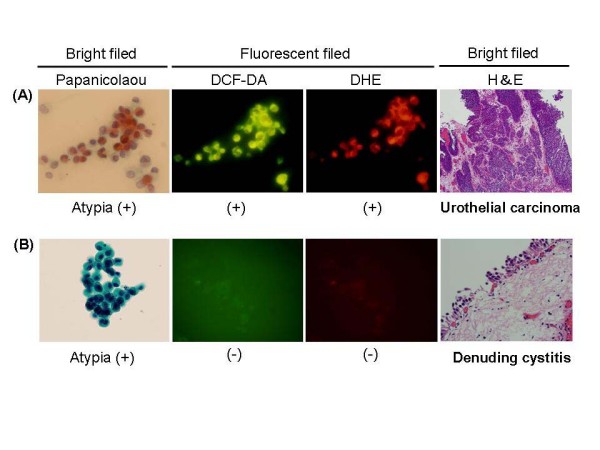
**Representative data of ROS urine cytology**. (A) Atypical urothelial cell cluster was found under bright field, and these cells were reactive to CM-DCFDA and DHE as determined under fluorescence filed (C-C is poitive and ROS-C is positive). Histological examination of bladder tumors (H&E) revealed superficially invasive/high grade urothelial carcinoma (pT1). (B) Cluster of atypical small sized urothelial cells were found under bright field, but intracellular ROS was not determined (C-C is 'suspicious' but ROS-C is negative). Histology of bladder specimen (H&E) revealed denuding cystitis without malignant findings.

Since the NOX4-ROS signal was strongly amplified in urothelial carcinoma cells, unlike in normal urothelial cells, we attempted to clarify the usefulness of ROS labeling in cytological diagnosis. Voided urine samples were collected from 50 different patients initially diagnosed with urothelial carcinomas of the urinary bladder (no previous history of bladder tumors) (Table 2) and 80 normal controls diagnosed with chronic cystitis, urinary tract stones or chronic pyelonephritis. Cells in urine samples were treated with the ROS-detecting fluorescent reagents, DCF-DA and DHE, and then routine Papanicolaou staining was performed. Initially, conventional cytological diagnosis (conventional cytology, C-C) was made and divided into 3 categories: negative, suspicious and positive. Then, we extracted ROS fluorescence-positive cells and examined whether they presented cytological atypia or whether cytologically atypical cells exhibit ROS generation under bright/fluorescent fields (termed ROS cytology, ROS-C). If the number of ROS-positive cells with significant atypia exceeded 20 cells, we judged it as a "positive finding of ROS-C." Figure 5A indicates typical positive cases: malignant cytological findings were observed by the Papanicolaou staining method under bright field microscopy; moreover, these cells were confirmed to generate superoxide anion and hydrogen peroxide under the fluorescence field. The histological diagnosis from transurethral resection of bladder cancer was high-grade, superficially invasive urothelial carcinoma (pT1). Figure 5B demonstrates the usefulness of ROS-C: atypical, small urothelial cells were detected by the Papanicolaou stain, but the number of atypical cell foci was too small with little atypia, which precluded a definite positive. Therefore, a diagnosis of "suspicious" was made. In contrast, the diagnosis by ROS-C was "negative" because we could not detect intracellular ROS production. Transurethral biopsy revealed no malignant denuding cystitis. As shown in Table 3, the sensitivities of ROS-C for low grade and non-invasive urothelial carcinomas were higher than those for C-C (75% and 77% vs. 35% and 39%, respectively) (P < 0.01), whereas there were no statistical differences for high-grade phenotypes between C-C and ROS-C (88% vs. 92%); the sensitivities for superficially or deeply invasive carcinomas and CIS were similar. Both C-C and ROS-C revealed high values for specificity (90% vs. 95%, no statistically significant difference). The positive predictive value (PPV) was 90.9% and negative predictive value (NPV) was 89.4%. Thus, ROS labeling together with C-C might improve accuracy by increasing the sensitivity for detecting bladder cancer, especially with low grade, non-invasive phenotypes. All 15 cases diagnosed as "suspicious" by C-C underwent cystoscopy examination. Of these, 10 cases were low-grade urothelial carcinomas and the others had normal urothelium featuring denuding cystitis etc. Interestingly, ROS-C had high sensitivity and specificity for cytologically "suspicious" cases at 90% and 85%, respectively.

### The use of ROS labeling in urine cytology for bladder cancer (follow-up cases with previous history of urothelial carcinomas)

Degeneration of urothelial cells would complicate cytological diagnosis even further in follow-up urine cytology after surgery and/or immunotherapy (such as Bacille de Calmette et Guérin (BCG) therapy, etc) for bladder cancer. We attempted to clarify the usefulness of ROS-C in follow-up urine cytology of bladder cancer. The characteristics of patients with a history of urinary bladder cancer are listed in Table 4. All patients had at least 1 follow-up cytoscopy examination after C-C and ROS-C, with a mean follow-up period of 4.7 months. As shown in Table 5, both the sensitivity and specificity of C-C at follow-up, particularly in cases with low-grade and non-invasive carcinoma recurrence were markedly decreased compared to those for cases initially diagnosed with urothelial carcinomas (low grade, 0% vs. 35%; non-invasive carcinoma, 0% vs. 39%). The sensitivity of C-C was very low even in cases with high-grade and invasive carcinomas or CIS (high grade, 57%; invasive carcinoma/carcinoma in situ, 47%). The sensitivities of ROS-C for low-grade and non-invasive carcinomas were 64% and 69%, respectively, which were statistically higher than those of C-C for cases initially diagnosed as urothelial carcinomas. The specificity of ROS-C was 97%, higher than that of C-C, which was 79% (P < 0.05). Thus, cytomorphological analysis of ROS-positive cells might be a reliable tool to improve the sensitivity of urine cytology with high specificity. The present data demonstrating zero sensitivity of urine cytology in low-grade urothelial carcinomas partly resulted from the small sample size. More data using adequate samples should be collected for definite conclusions. The 14 cases cytologically diagnosed as 'suspicious' consisted of 7 cancer cases (2 were high-grade and 5 were low-grade carcinomas) and the remaining 7 cases featured normal urothelium, including post-BCG granulomatous cystitis. The sensitivities of ROS-C for low and high-grade urothelial carcinomas were 60% and 100%, respectively for these "suspicious" cases, whereas specificity was 100%. Thus, ROS-C could determine whether cases diagnosed as "suspicious" by C-C are truly positive or negative.

**Table 4:** Characteristics of follow-up cases after urothelial carcinoma

Age (years)	73.1 (54-90)
Gender (M:F)	52:10
Negative	34
Positive	28
Stage	
pTa	13
pT1	6
≧pT2	3
pTis	6
Grade	
Low grade	14
High grade	14

**Table 5:** Comparison of conventional cytology with ROS cytology (follow-up cases with previous history of urothelial carcinomas)

	Conventional cytology				ROS cytology		
Urothelial carcinoma	Positive	Suspicious	Negative	Sensitivity	Positive	Negative	Sensitivity
Low grade(n = 14)	0	5	9	0%	9	5	64%
High grade(n = 14)	8	2	4	57%	14	0	100%
Non-invasive papillary	0	5	8	0%	9	4	64%
(n = 13)							
Superficially invasive	3	1	2	50%	5	1	83%
(n = 6)							
Deep invasive	1	1	1	33%	3	0	100%
(n = 3)							
Carcinoma in situ	3	1	2	50%	6	0	100%
(n = 10)							
				Specificity			Specificity
Negative(n = 34)	0	7	27	79%	1	33	97%

## Discussion

This is the first study showing that the NOX4-ROS signal contributes to the survival of human urothelial carcinoma cells via progression of the G1/S transition. This study also shows the practical use of ROS labeling in urinary cytology. Previously, we showed that a novel isoform of the DNA repair enzyme ALKBH, ALKBH-8, contributes to progression of urothelial carcinoma cells via NOX1-ROS signal-mediated resistance to apoptosis induction [14]. Although ROS was produced in cancer cells irrespective of pathological grade or stage, immunohistochemistry showed high expression of NOX1 proteins in high-grade, superficially, and deeply invasive carcinomas (pT1 and > pT2), as well as in carcinoma in situ, but not in low-grade and non-invasive phenotypes (pTa). The present study uncovered key data for the resolution of this problem and provided a novel mechanism involved in the development of bladder cancer: NOX4 was highly expressed in near-equivalent levels in low and high grade or non-invasive and invasive urothelial carcinomas, including dysplasia, but not in normal urothelium. Moreover, NOX4 silencing reduced ROS generation and suppressed cancer cell growth via p16-dependent cell cycle arrest at the G1 phase, both in vitro and in vivo. These data indicate that NOX4-mediated ROS generation contributes to an early step in urothelial carcinogenesis and cancer cell survival. In addition to NOX4, NOX1-mediated enhancement of ROS generation might result in bladder cancer cells of a more aggressive phenotype. Dysplasia represents an early morphologic manifestation of progressive alteration between normal urothelium and carcinoma in situ, and clinicians must be attentive to the clinical development. In contrast to CIS, clinical therapy is not directly indicated for dysplasia. Therefore, pathologists should differentiate dysplasia from other flat atypical urothelial lesions. There have been a number of reports regarding molecular markers that enable accurate differential diagnosis of flat atypical urothelial lesions. CK20 and CD44 are the most useful markers for distinguishing atypia of unknown significance (AUS) from dysplasia (for example, CK20 expression is usually limited to the umbrella cells but is expressed in the deeper mucosal layer in AUS) and p53 immunostains typically highlight the dysplastic cells [19-22]. However, Murata et al. [19] demonstrated that molecular and immunohistochemical analyses, including fluorescence in situ hybridization (FISH), to detect expression of the high molecular weight cytokeratin, Ki-67 and p53 can discriminate between neoplastic and non-neoplastic lesions. However, they cannot reliably resolve the diagnostic variation of flat intraepithelial lesions. Immunohistochemical analysis of NOX4, in addition to these markers, will enable differential diagnosis of dysplasia. NOX4 knockdown was expected to have no significant effects on NOX1 expression but ROS levels were strongly decreased, and the same phenomenon was also observed in a NOX1 knockdown experiment (data not shown). Therefore, in addition to NOX1, NOX4 is required for the maintenance of intracellular ROS in urothelial carcinoma cells. NOX4 should therefore be the target molecule in bladder cancer treatment. Since the expression of NOX4 was relatively high not only in urothelial carcinoma cells but also in dysplasia as precancerous lesions, we hope to develop prophylaxis against bladder cancer occurrence and recurrence with a new strategy focusing on the NOX4-ROS signal. Various studies have been carried out on the role of NOX4 in pancreatic cancer progression. Mochizuki et al. demonstrated inhibition of NOX4 activates apoptosis via the Akt/apoptosis signal-regulating kinase 1 pathway in pancreatic cancer PANC-1 cells [20], but apoptosis was not observed in urothelial carcinoma cells by NOX4 knockdown, unlike in NOX1 gene silencing [14]. The biological and clinical significance of NOX may be specific to NOX isoforms, cell types, and/or a combination of NOX family members.

We demonstrated here for the first time the validity of ROS labeling for urinary cytological diagnosis. The advantages of urine cytology include high specificity; it is an established technique and minimal sample processing is required. In contrast, the high level of expertise required, significant interobserver variation, and low sensitivity (especially for low-grade phenotypes) are considered disadvantages [8,21,22]. At present, several biomarkers are commercially available: the bladder tumor antigen (BTA) test measures urine levels of H-related protein, which is similar to H protein and is secreted in high levels by tumor cells. The BTA test outperforms cytology in sensitivity, but its specificity falls below that of cytology because of false positive results by inflammatory or infectious conditions [23,24]. Nuclear matrix protein (NMP) 22, a regulator of mitosis, is known to be increased in malignant urothelium, and quantitative immunoassay of NMP22 demonstrates superior sensitivity for bladder cancer compared to urine cytology [25]. However, the specificity is generally lower than that of urine cytology due to NMP22 dependency on the cut-off points used [26]. Lately, FISH has attracted considerable interest for its high sensitivity (69-96%) and specificity (65-96%) [27]. FISH identifies common alterations in the chromosomal copy number (chromosomes 3, 7, and 17) and loss of the 9p21 locus, and is largely unaffected by inflammatory conditions. Recent studies have shown that FISH could detect subclinical neoplastic changes [28,29]. However, FISH analysis of urine specimens requires specialized laboratory equipment, expensive reagents, and a high level of expertise; therefore, it is not suited to high throughput testing. The International and European guideline panels no longer recommend the use of FISH tests, including UroVysion, owing to inferior specificity and a lack of reliable prospective testing [1,30]. A fault common to all of these tests is that they cannot make full use of the merits of urine cytology. A new diagnostic system that counteracts the low sensitivity of cytology but retains its high specificity should be established. Our idea was to introduce labeling of ROS produced by urothelial carcinoma cells (but not by non-malignant cells) to conventional cytology: ROS-positive cells are theoretically considered to be malignant cells despite little morphological atypia. The present study clearly indicates that extraction of ROS-producing cells using the fluorescent dyes CM-DCFDA and DHE statistically improves the accuracy of urine cytology. Moreover, this system was not affected by post-surgical or therapy-related inflammation or degeneration. Sometimes, pathologists and cytologists face the problem of under- or over-diagnosis, and the number of suspicious cases that cannot be identified as malignant or benign increase due to degenerative morphological changes (for example, the size of malignant cells are miniaturized, or normal cells are distended and exhibit atypia). This problem tends to occur especially in follow-up urine samples after surgical or immune therapy. ROS-C, available in such cases, demonstrated high sensitivity and high specificity. Since the fluorescence level of ROS-reactive dyes was much higher than that of non-specific self-fluorescence, there were very few ROS-C false-positive results in the current study. Inflammatory cells such as neutrophils and macrophages were labeled by ROS dyes but were distinguished from epithelial tumor cells by cytomorphological characteristics. Inter- and intra-observer variabilities are worth considering for cytologists who lack experience in handling urine samples and/or fluorescence microscopy. We are trying to construct more objective rules to help decide whether ROS positive cells are malignant--for example, by setting a threshold of fluorescence intensity.

## Conclusion

NOX4 contributes to ROS production and cell survival in human urothelial carcinoma cells. From the immunoprofile of NOX4 in normal, dysplastic, and malignant urothelial cells, NOX4 might be involved in an early stage of urothelial carcinogenesis and be a potential new molecular target for bladder cancer therapy. Moreover, fluorescence labeling of ROS can bring about accurate pathological and cytological diagnoses of human bladder cancers. Conventional urine cytology exhibits high specificity, but unfortunately, low sensitivity, to detect bladder cancer. In contrast, ROS labeling methods alone could exhibit high sensitivity but not high specificity. In this study, we added ROS labeling methods to conventional cytology and created a new assay system with high specificity and sensitivity.

## Competing interests

The authors declare that they have no competing interests.

## Authors' contributions

KS and TF carried out cell bioloigical analyses using human urothelial carcinoma cell lines and drafted the manuscript. SA and KF carried out the animal experiments. KS and NK participated in cytological diagnosis/analysis using human urine samples. KS and SA participated in the design of the study and performed the statistical analysis. NK conceived of the study, and participated in its design and coordination. All authors read and approved the final manuscript.

## Pre-publication history

The pre-publication history for this paper can be accessed here:

http://www.biomedcentral.com/1471-2490/11/22/prepub
